# Development and Validation of a Prediction Model for Cardiovascular Events in Exercise Assessment of Coronary Heart Disease Patients After Percutaneous Coronary Intervention

**DOI:** 10.3389/fcvm.2022.798446

**Published:** 2022-04-26

**Authors:** Tao Shen, Chuan Ren, Wei Zhao, Liyuan Tao, Shunlin Xu, Chengduo Zhang, Wei Gao

**Affiliations:** ^1^Department of Cardiology, Peking University Third Hospital, NHC Key Laboratory of Cardiovascular Molecular Biology and Regulatory Peptides, Key Laboratory of Molecular Cardiovascular Science, Ministry of Education, Beijing, China; ^2^Physical Examination Center, Peking University Third Hospital, Beijing, China; ^3^Research Center of Clinical Epidemiology, Peking University Third Hospital, Beijing, China

**Keywords:** coronary heart disease, prediction model, cardiac rehabilitation, exercise risk, exercise safety

## Abstract

**Objective:**

This study aimed to develop a model for predicting cardiovascular events in the exercise assessment of patients with coronary heart disease after percutaneous coronary intervention (PCI) based on multidimensional clinical information.

**Methods:**

A total of 2,455 post-PCI patients who underwent cardiopulmonary exercise testing (CPET) at the Peking University Third Hospital from January 2016 to September 2019 were retrospectively included in this study; 1,449 post-PCI patients from January 2018 to September 2019 were assigned as the development cohort; and 1,006 post-PCI patients from January 2016 to December 2017 were assigned as the validation cohort. Clinical data of patients before testing and various indicators in the exercise assessment were collected. CPET-related cardiovascular events were also collected, including new-onset angina pectoris, frequent premature ventricular contractions, ventricular tachycardia, atrial tachycardia, and bundle branch block during the examination. A nomogram model for predicting CPET-related cardiovascular events was further developed and validated.

**Results:**

In the development cohort, the mean age of 1,449 post-PCI patients was 60.7 ± 10.1 years. CPET-related cardiovascular events occurred in 43 cases (2.9%) without fatal events. CPET-related cardiovascular events were independently associated with age, glycosylated hemoglobin, systolic velocity of mitral annulus, ΔVO_2_/ΔWR slope inflection, and VE/VCO_2_ slope > 30. The C-index of the nomogram model for predicting CPET-related cardiovascular events was 0.830, and the area under the ROC curve was 0.830 (95% CI: 0.764–0.896). For the validation cohort of 1,006 patients, the area under the ROC curve was 0.807 (95% CI: 0.737–0.877).

**Conclusion:**

Post-PCI patients with older age, unsatisfactory blood glucose control, impaired left ventricular systolic function, oxygen uptake parameter trajectory inflection, and poor ventilation efficiency have a higher risk of cardiovascular events in exercise assessment. The nomogram prediction model performs well in predicting cardiovascular events in the exercise assessment of post-PCI patients and can provide an individualized plan for exercise risk prediction.

## Introduction

Percutaneous coronary intervention (PCI) has become the most important method of revascularization for patients with coronary heart disease (CHD), which not only effectively improves the clinical symptoms of patients but also greatly reduces the mortality of patients (1). However, most patients still suffer from decreased exercise tolerance and mental problems such as anxiety and depression after surgery. Exercise-based cardiac rehabilitation is an important component of secondary prevention for CHD, which can significantly reduce mortality rate, rehospitalization rate, and revascularization rate; promote physical and mental health; and improve the quality of life of patients with CHD (2, 3).

Improper exercise may induce an increased cardiovascular risk. In order to ensure the safety and efficiency of exercise, patients after PCI should receive appropriate exercise risk assessment before participating in exercise training of a specific intensity. By comprehensively applying respiratory gas monitoring and treadmill exercise or bicycle ergometry technology, cardiopulmonary exercise testing (CPET) can detect the dynamic changes in oxygen uptake, carbon dioxide output, blood pressure, electrocardiogram (ECG), and other indicators under the stimulation of different workloads (4). Real-time analysis of those indicators during testing makes it possible to provide an immediate prediction of cardiovascular events in exercise assessment and to further avoid risks by terminating exercise testing in time. Therefore, exercise-based cardiac rehabilitation guided by CPET is safer and conducive to avoiding exercise risks and can enhance cardiovascular benefits and exercise safety (5, 6).

Previous studies have shown that exercise assessment is relatively safe in patients with CHD, but few studies have been done on risk prediction. Targeting post-PCI patients and including clinical indicators before testing and CPET indicators obtained during exercise, this study developed a model for predicting the risk of exercise-related cardiovascular events.

## Materials and Methods

### General Information

We retrospectively included 2,455 patients with CHD who underwent PCI at the Peking University Third Hospital, followed by CPET from January 2016 to September 2019. Inclusion criteria were as follows: age over 18 years, clinical diagnosis of CHD and receiving PCI treatment, and undergoing CPET for the first time within 6 months after PCI. Exclusion criteria were as follows: severe cardiac insufficiency, New York Heart Association (NYHA) functional class III–IV, complicated with valvular heart disease and cardiomyopathy, complicated with severe lung disease and respiratory failure, complicated with severe hepatic and renal insufficiency, complicated with malignant tumor and hematological disease, and difficulty in physical activity.

A total of 1,449 post-PCI patients who underwent CPET from January 2018 to September 2019 were assigned as the development cohort; 1,006 post-PCI patients who underwent CPET from January 2016 to December 2017 were included in the validation cohort. Clinical data of patients before testing were collected and analyzed, including blood test, echocardiography, and coronary angiography information within 3 months before testing.

### Cardiopulmonary Exercise Testing

Ultima CardiO2 CPET equipment (Medical Graphics Corporation, St Louis, MO, United States) was used. All patients adopted Ramp protocol with cycle ergometer; the initial power output is usually 10 W (60 kpm/min), followed by increases of 25 W every 2 or 3 min until end points are reached. ECG, blood pressure, and systemic responses of patients were monitored during exercise. Patients were encouraged to perform symptom-restricted exercise (BORG rating of perceived exertion scale > 18) and respiratory exchange rate (RER) ≥ 1.1. After the exercise testing, all patients were observed for 5 min under no workload. The whole process was carried out under 12-lead ECG monitoring and accompanied by professional physicians. All testing results were analyzed, from which valid data were adopted.

During the testing, ECG, blood pressure, gas exchange, and other information of patients were continuously collected and recorded. Parameters directly measured included peak oxygen uptake (VO_2_peak), peak heart rate, peak oxygen pulse, peak work rate, oxygen uptake at anaerobic threshold, heart rate at anaerobic threshold, oxygen pulse at anaerobic threshold, and work rate at anaerobic threshold. They were further combined to obtain the following parameters (4): inflection of oxygen uptake relative to work rate (ΔVO_2_/ΔWR slope inflection), oxygen pulse flattening, minute ventilation/carbon dioxide output slope (VE/VCO_2_ slope), oxygen uptake efficiency slope (OUES). ΔVO_2_/ΔWR slope is the increasing slope of oxygen uptake relative to work rate, reflecting the increase in the magnitude of oxygen uptake per unit work rate. When the curve of oxygen uptake relative to work rate shows an inflection point, it is called the ΔVO_2_/ΔWR slope inflection. Under normal circumstances, the oxygen pulse curve shows an upward trend during exercise. When the oxygen pulse does not increase with exercise intensity and the curve shows a horizontal or even downward trend, oxygen pulse flattening occurs. VE/VCO_2_ slope is a slope calculated by linear regression based on minute ventilation and carbon dioxide output during the entire exercise process. OUES is obtained by fitting a logarithmic curve of ventilation and oxygen uptake during exercise. It represents the increased rate of oxygen uptake relative to per minute ventilation.

Data on CPET-related cardiovascular events were collected. According to the scientific statement from American Heart Association on exercise standards for testing and training (7), CPET-related cardiovascular events can be classified into the following situations: (1) angina pectoris, referring to typical ischemic chest pain accompanied by ischemic ECG changes; (2) premature ventricular contractions (PVCs), including frequent PVCs (more than 5 beats per minute), multisource PVCs, and trigeminy PVCs during exercise or recovery; (3) atrial arrhythmia, referring to frequent premature atrial contractions (more than 5 beats per minute), atrial tachycardia, atrial flutter, atrial fibrillation, and paroxysmal supraventricular tachycardia during exercise or recovery; (4) sustained or non-sustained ventricular tachycardia (NSVT); (5) bradyarrhythmia, including new-onset sinus arrest, atrioventricular block, and left/right bundle branch block during the examination; (6) drop in blood pressure during exercise of more than 10 mmHg, accompanied by evidence of ischemia; and (7) other cardiovascular events leading to the termination of CPET. The standard criterion for positive ECG response is horizontal or downsloping ST-segment depression of ≥ 0.10 mV (1 mm) for 80 ms. The development of > 0.10 mV of J-point elevation that is persistently elevated (> 0.10 mV) at 60 ms after the J point in 3 consecutive beats with a stable baseline is also considered an abnormal response (8).

### Statistical Methods

Statistical analysis was performed using SPSS 20.0. Categorical data were presented by case number and percentage (%), and the comparison between groups was conducted by a χ*^2^* test. A normal distribution test was performed for measurement data. Those with normal distribution were presented by mean ± standard deviation (mean ± SD), and the comparison between groups was conducted by independent samples *t*-test. Non-normally distributed measurement data were presented by median (first quartile, third quartile) [median (Q1, Q3)], and the comparison between groups was conducted by the Mann–Whitney U test. Multivariate linear regression analysis was used for multivariate analysis. *P* < 0.05 was taken as a statistically significant difference. All *P*-values were two-tailed. The multiplicity was not corrected. In the data set of independent variables, some data were incomplete, with less than 3% of missing data for included features. Based on the patient data that were complete, we replaced missing values with the mean value of corresponding variables. Lasso regression was performed for clinical indicators before testing, and CPET indicators were obtained during exercise using R language. Variables with statistical significance in Lasso regression were included in the logistic regression model. Based on the correlation coefficient of variables in the logistic regression model, a nomogram model for predicting CPET-related cardiovascular events was developed and validated. The Hosmer–Lemeshow test was used to compare the observed and predicted probabilities. There were 12 subgroups used. The statement was added when reporting the chi-squared results.

## Results

### General Information of Patients

General information of 1,449 patients with CHD after PCI in the development cohort was analyzed (Table 1). The mean age of patients was 60.7 ± 10.1 years, and 1,151 (79.4%) patients were male patients; 1,208 (83.4%) patients had at least one risk factor of CHD (including hypertension, diabetes, hyperlipidemia, and smoking); and 417 (28.7%) patients had a history of myocardial infarction. The mean time after myocardial infarction was 3.4 ± 1.2 months in patients with myocardial infarction. There were 119 cases of anterior wall infarction (28.5%), 121 cases of inferior wall infarction (29.0%), 26 cases of lateral or posterior wall infarction (6.1%), and 151 cases of non-ST-segment elevation myocardial infarction (36.2%). Complete revascularization was performed in 991 patients (68.3%); 185 patients (12.7%) were in NYHA class II.

**TABLE 1:** Clinical information of patients with CHD after PCI in the development cohort.

Parameters	Total (%), mean ± SD
Age (years)	60.7 ± 10.1
Male, *N* (%)	1151(79.4)
BMI (Kg/m^2^)	25.9 ± 3.7
History of myocardial infarction, *N* (%)	417(28.7)
Complete revascularization, *N* (%)	991(68.3)
NYHA functional class II, *N* (%)	185(12.7)
Hypertension, *N* (%)	895(61.7)
Diabetes, *N* (%)	478(37.9)
Hyperlipidemia, *N* (%)	995(68.9)
Smoking history, *N* (%)	689(47.5)
Family history of CHD, *N* (%)	431(29.7)
Exercise habit, *N* (%)	948(66.4)

*BMI, body mass index.*

### Development of the Prediction Model for Cardiopulmonary Exercise Testing-Related Cardiovascular Events in Patients With Coronary Heart Disease After Percutaneous Coronary Intervention

In the development cohort, CPET-related cardiovascular events occurred in 43 (2.9%) patients out of 1,449 patients with CHD after PCI, which were specifically classified as follows: angina pectoris in 13 cases, frequent PVCs in 14 cases, NSVT in 4 cases, atrial tachycardia in 9 cases, and bradyarrhythmia arrhythmia in 3 cases. There were no fatal incidents.

Taking 1,449 patients with CHD after PCI as the development cohort, a total of 32 variables were included for analysis, including baseline characteristics, laboratory indicators, echocardiography indicators, and CPET indicators obtained during exercise. Compared with the group without CPET-related cardiovascular events, those with cardiovascular events were older (66.2 ± 10.2 vs. 60.5 ± 10.2, *P* = 0.002), had a higher proportion of cases in terms of the history of myocardial infarction (42.4 vs. 28.3%, *P* = 0.038), and had a complication of diabetes (48.8 vs. 32.3%, *P* = 0.012) (Table 2). They had higher level of glycosylated hemoglobin (HbA1c) (7.2 ± 0.9% vs. 6.6 ± 1.2%, *P* = 0.001), and NT-proBNP [105.5 (50.2, 370.6) pg/ml vs. 91.3 (39.3, 229.0) pg/ml, *P* = 0.018] had lower peak systolic velocity of mitral annulus (Sm) (8.5 ± 2.0 cm/s vs. 10.2 ± 2.3 cm/s, *P* = 0.008). Besides, in the group with cardiovascular events, there were a higher proportion of cases with positive ECG in exercise testing (32.6 vs. 10.3%, *P* < 0.001), ΔVO_2_/ΔWR slope inflection (23.3 vs. 6.4%, *P* < 0.001), and oxygen pulse flattening (37.2 vs. 15.1%, *P* < 0.001). They also had larger VE/VCO_2_ slope (32.1 ± 4.9 vs. 29.7 ± 5.2, *P* = 0.007) and lower VO_2_ peak (17.6 ± 4.5 ml/min/kg vs. 18.6 ± 5.1 ml/min/kg, *P* < 0.001).

**TABLE 2:** Baseline characteristics of patients with CHD after PCI grouped by CPET-related cardiovascular events.

Baseline characteristics	With CPET-related cardiovascular events (*n* = 43)	Without CPET-related cardiovascular events (*n* = 1406)	*P-*value
Age (years)	66.2 ± 8.4	60.5 ± 10.2	0.002
Male, *N* (%)	38 (88.4)	1113 (79.2)	0.180
BMI (Kg/m2)	25.8 ± 3.7	25.8 ± 3.5	0.901
History of myocardial infarction, *N* (%)	19 (42.4)	398 (28.3)	0.038
Complete revascularization, *N* (%)	32 (74.4)	959 (68.2)	0.505
NYHA functional class II, *N* (%)	8 (18.6)	177 (12.6)	0.245
Hypertension, *N* (%)	25 (58.1)	870 (61.9)	0.635
Hyperlipidemia, *N* (%)	34 (79.1)	961 (68.3)	0.181
Diabetes, *N* (%)	21 (48.8)	454 (32.3)	0.012
Smoking history, *N* (%)	22 (51.2)	659 (46.9)	0.643
Family history of CHD, *N* (%)	16 (37.2)	415 (29.5)	0.310
Exercise habit, *N* (%)	26 (60.5)	922 (65.6)	0.516
**Laboratory indicators**			
HbA1c (%)	7.2 ± 0.9	6.6 ± 1.2	0.001
Cr (μmol/L)	91.0 ± 11.4	89.0 ± 20.8	0.624
LDL (mmol/L)	2.3 ± 0.7	2.0 ± 0.7	0.052
Hb (g/L)	139.7 ± 17.5	141.2 ± 13.9	0.607
NT-proBNP (pg/mL)	105.5 (50.2, 370.6)	91.3 (39.3, 229.0)	0.018
**Echocardiography indicators**			
LVEDD (mm)	48.6 ± 7.0	48.9 ± 15.0	0.932
LVEF (%)	63.2 ± 14.6	65.6 ± 11.0	0.401
Sm (cm/s)	8.5 ± 2.0	10.2 ± 2.3	0.008
E/Em	8.7 ± 3.5	8.0 ± 3.0	0.316
LAA (cm^2^)	18.5 ± 3.9	19.0 ± 3.7	0.555
**CPET indicators**			
Positive ECG, *N* (%)	14 (32.6)	145 (10.3)	< 0.001
ΔVO_2_/ΔWR slope inflection, *N* (%)	10 (23.3)	90 (6.4)	< 0.001
Oxygen pulse flattening, *N* (%)	16 (37.2)	213 (15.1)	< 0.001
VO_2_@AT (ml/min/kg)	11.4 ± 3.5	11.9 ± 3.4	0.058
HR@AT (bpm)	102 ± 12	102 ± 14	0.836
VO_2_peak (ml/min/kg)	17.6 ± 4.5	18.6 ± 5.1	< 0.001
HRpeak (bpm)	125 ± 20	126 ± 20	0.748
SBPpeak (mmHg)	167 ± 27	169 ± 28	0.690
VE/VCO_2_ slope > 30	31 (72.1)	518 (36.8)	< 0.001
OUES	1520.8 ± 338.4	1574.2 ± 415	0.453

*BMI, body mass index; HbA1c, glycosylated hemoglobin A1c; Cr, creatinine; LDL, low-density lipoprotein; Hb, hemoglobin; NT-proBNP, N-terminal pro-brain natriuretic peptide; LVEDD, left ventricular end-diastolic dimension; LVEF, left ventricular ejection fraction; Sm, systolic velocity of mitral annulus; E/Em, the ratio of early diastolic transmitral flow velocity to early diastolic tissue velocity; LAA, left atrium area; VO_2_@AT, oxygen uptake at anaerobic threshold; HR@AT, heart rate at anaerobic threshold; VO_2_peak, peak oxygen uptake; HRpeak, peak heart rate; SBPpeak, peak systolic blood pressure; VE/VCO_2_ slope, ventilation per carbon dioxide output slope; OUES, oxygen uptake efficiency slope.*

**FIGURE 1:**
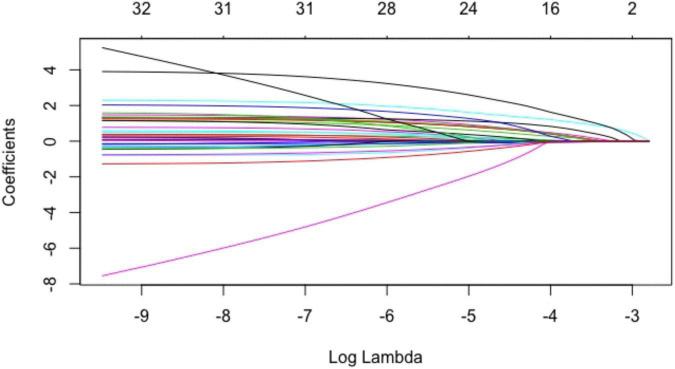
Coefficients of baseline variables in the Lasso regression. Each curve in the figure represented the trajectory of each independent variable coefficient. The ordinate was the value of the coefficient, and the lower abscissa was log(λ). The upper abscissa was the number of coefficients in the model. According to different λ values, individual coefficients without a coefficient value of 0 were variables included in the model.

**FIGURE 2:**
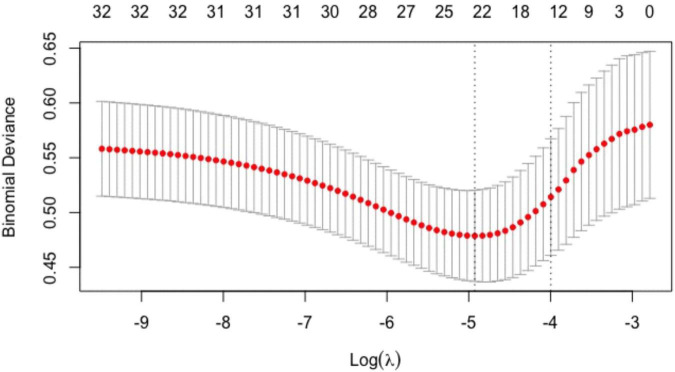
Tuning parameter (λ) selection in the Lasso regression model. Models were fitted using cross-validation, and then, the optimal λ value was selected to have a more accurate estimate of the performance of the model. For each λ value, around the mean of the target parameter shown in the red dot, we could obtain a confidence interval of the target parameter. The two dashed lines indicated two particular λ values, namely, the one with the smallest model error, and the one with the best model performance but the smallest number of independent variables.

The above baseline variables were included in the Lasso regression. Results showed that 12 variables, namely, age, male, smoking history, history of myocardial infarction, NYHA class II, exercise habits, HbA1c, Sm, positive ECG in exercise testing, ΔVO_2_/ΔWR slope inflection, oxygen pulse flattening, and VE/VCO_2_ slope > 30, had significant predictive value (Figures 1, 2).

**FIGURE 3:**
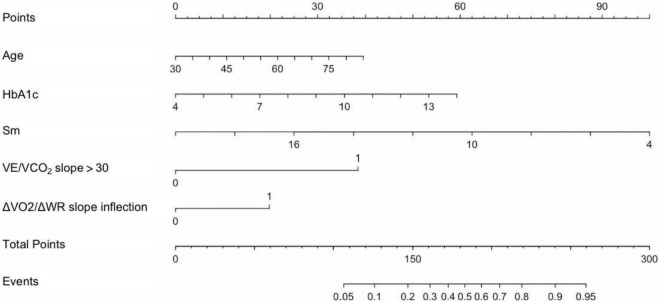
Nomogram model for predicting CPET-related cardiovascular events in patients with CHD after PCI. The first line was the points of risk factors, and the second to sixth lines were risk factors. By summarizing each risk factor point, total points could be obtained. Then, the event probability (line 8) was calculated according to the total points of line 7. HbA1c, glycosylated hemoglobin A1c; Sm, systolic velocity of mitral annulus; VE/VCO_2_ slope > 30, minute ventilation/carbon dioxide output slope > 30; ΔVO_2_/ΔWR slope inflection, inflection of oxygen uptake relative to work rate; Events, CPET-related cardiovascular events.

Multivariate logistic regression analysis was conducted with cardiovascular events as dependent variables and the above 12 variables as independent variables. Results showed that age [OR 1.039 (95% CI: 1.002–1.078), *P* = 0.040], HbA1c [OR 1.346 (95% CI: 1.055–1.717), *P* = 0.017], Sm [OR 0.795 (95% CI: 0.678–0.931), *P* = 0.004], ΔVO_2_/ΔWR slope inflection [OR 4.796 (95% CI: 1.986–10.687), *P* = 0.001], and VE/VCO_2_ slope > 30 [OR 1.976 (95% CI: 1.001–3.987), *P* = 0.047] were independently associated with CPET-related cardiovascular events. Based on the correlation coefficients of pretesting clinical indicators and CPET indicators in the logistic regression model, a nomogram model was developed for predicting CPET-related cardiovascular events (Figure 3).

**FIGURE 4:**
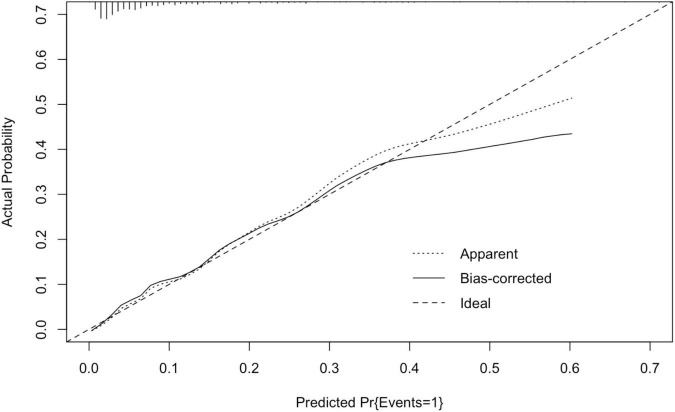
Calibration curve of nomogram model in the development cohort. The standard curve was a straight line passing through the origin of the coordinate axis. If the predicted calibration curve is closer to the standard curve, the better the prediction ability of the nomogram. “Bias-corrected” was the correction curve, while “Ideal” was the standard curve. The graphical predicted and measured values are basically consistent, indicating that the nomogram model of this study has a good calibration.

The area under the ROC curve of the nomogram model was 0.830 (95% CI: 0.764–0.896). The predictive effect of the model was validated using the Bootstrap internal validation method and based on the modeling data. C-index was calculated to be 0.830, indicating that the predictive ability of the nomogram was relatively accurate. Results of Hosmer–Lemeshow test (χ*^2^* = 3.098, *P* > 0.05) suggested that there was no statistically significant difference between the predicted probability of the model and the actual observed probability. As demonstrated in the nomogram calibration curve, the predicted value was basically consistent with the measured value, indicating that the nomogram model in this study had good calibration (Figure 4).

### Validation of the Prediction Model for Cardiopulmonary Exercise Testing-Related Cardiovascular Events in Patients With Coronary Heart Disease After Percutaneous Coronary Intervention

The developed prediction model for CPET-related cardiovascular events in patients after PCI was further validated. A total of 1,006 patients with CHD who underwent CPET after PCI from January 2016 to December 2017 were selected as the validation cohort. The mean age was 60.6 ± 10.2 years and 801 (79.6%) were male patients; 923 (91.7%) patients had at least one risk factor of CHD (including hypertension, diabetes, hyperlipidemia, and smoking); 325 (32.3%) patients had a history of myocardial infarction; 719 (71.5%) patients underwent complete revascularization; and 212 (21.0%) patients were in NYHA class II.

In the validation cohort, CPET-related cardiovascular events occurred in 38 (3.7%) patients, which were classified specifically as follows: angina pectoris in 8 cases, frequent PVCs in 10 cases, NSVT in 6 cases, atrial tachycardia in 10 cases, and bradyarrhythmia arrhythmia in 4 cases. There were no fatal incidents.

The developed nomogram model for predicting CPET-related cardiovascular events in patients with CHD after PCI included variables of age, HbA1c, Sm, ΔVO_2_/ΔWR slope inflection, and VE/VCO_2_ slope. In the validation cohort, compared with non-event group, those with CPET-related cardiovascular events were older (66.1 ± 10.7 vs. 60.4 ± 10.0, *P* = 0.001), had higher level of HbA1c (7.4 ± 2.0% vs. 6.6 ± 2.0%, *P* = 0.001), and had lower Sm (9.0 ± 1.7 cm/s vs. 10.3 ± 2.3 cm/s, *P* = 0.001). There were also a higher proportion of cases with ΔVO_2_/ΔWR slope inflection (23.7 vs. 5.8%, *P* < 0.001) and VE/VCO_2_ slope > 30 (52.6 vs. 36.0%, *P* = 0.040).

**FIGURE 5:**
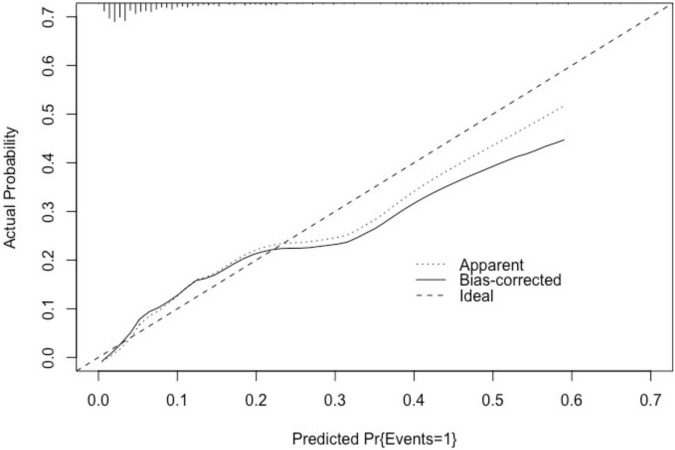
Calibration curve of nomogram model in the validation cohort. As demonstrated in the calibration curve of the nomogram model in the validation cohort, the predicted value was basically consistent with the measured value. “Basically consistent” means that the actual solid line and the ideal dashed line were basically parallel, indicating that the nomogram model of this study has a good calibration.

The above indicators were used to predict the risk of exercise testing for patients in the validation cohort. The predicted probability of the nomogram was taken as the state variable, and the actual probability of CPET-related cardiovascular events was taken as the test variable to compare the difference between the predicted result and the actual observation result. The area under the ROC curve of the nomogram model in the validation cohort was 0.807 (95% CI: 0.737–0.877), indicating the good discrimination ability of the prediction model. Results of Hosmer–Lemeshow goodness-of-fit test (χ*^2^* = 3.517, *P* > 0.05) suggested that there was no statistical significance between the predicted probability and the actual probability. The degrees of freedom was 11. As demonstrated in the calibration curve of the nomogram model in the validation cohort, the predicted value was basically consistent with the measured value, indicating that the nomogram model had good calibration for the validation cohort (Figure 5).

## Discussion

This study developed a model for predicting cardiovascular events in the exercise assessment of post-PCI patients based on multidimensional clinical information. The nomogram prediction model performs well in predicting cardiovascular events in the exercise assessment. Post-PCI patients with older age, abnormal blood glucose, impaired left ventricular systolic function, and abnormal CPET gas indicators (oxygen uptake parameter trajectory inflection and poor ventilation efficiency) have a higher risk of cardiovascular events in exercise assessment.

### Variable Selection and Development of the Prediction Model Using Lasso Regression

Lasso regression is characterized by variable selection and complexity regularization while fitting the generalized linear model. Variable selection refers to selectively including variables into the model to get parameters with better performance. Complexity regularization refers to controlling the complexity of the model through parameters to avoid over-fitting. For the linear model, the complexity of a model is directly related to the number of variables included. The more variables the model includes, the higher the complexity will be. It also increases the danger of over-fitting. In Lasso regression, the degree of complexity regularization is controlled by the parameter λ. The larger the λ is, the greater the penalty against complexity in the multivariable linear regression model will be, so as to finally obtain a model with fewer variables (9). Using Lasso regression, this study included various baseline variables of post-PCI patients. The developed nomogram model could effectively identify exercise-related events. The area under the ROC curve of the model was 0.830 (95% CI: 0.764–0.896), indicating the important value of the prediction model in identifying CPET-related cardiovascular events.

### Importance of Evaluating Blood Glucose Control

This study found that patients with elevated HbA1c had a higher risk of cardiovascular events during exercise testing, regardless of their history of diabetes. Some evidence suggests that diabetes may be associated with exercise-related cardiovascular events (10). A possible mechanism may be that long-term hyperglycemia leads to microangiopathy, autonomic nervous dysfunction, energy metabolism disorders, etc., which further causes ventricular diastolic dysfunction and cardiac chronotropic incompetence (11). Considering the potential risks of exercise in patients with diabetes, ESC recommends that all patients with diabetes should perform cardiovascular safety assessment before exercise, including blood glucose control, complications, and treatment regimens. Those asymptomatic patients with normal results in cardiovascular assessment and exercise testing can participate in the exercise of various intensities (12).

### Significance of Gas Metabolism Indicators During Exercise

Real-time analysis of indicators during testing makes it possible to provide an immediate prediction of cardiovascular events in exercise assessment and to further avoid risks by terminating exercise testing in time.

Under normal physiological conditions, oxygen uptake relative to work rate (ΔVO_2_/ΔWR) shows a linear increase with a slope of about 10 ml/min/watt. Abnormal ΔVO_2_/ΔWR slope usually reflects the exercise restriction in the pathological state of CHD. There may be no abnormality of VO_2_/ΔWR trajectory before the myocardial ischemic threshold. However, when exercise induces acute cardiac events, cardiac output decreases and the ΔVO_2_/ΔWR slope will have an inflection point or even drop sharply, which is easy to be identified by the tester (13, 14). Abnormal ΔVO_2_/ΔWR trajectory has an important diagnostic value for obstructive CHD (15). Myocardial ischemia provides reference for the diagnosis of myocardial ischemia. This study showed that the ΔVO_2_/ΔWR slope inflection was an independent risk factor for CPET-related cardiovascular events in patients after PCI. Belardinelli et al. have reported that the ΔVO_2_/ΔWR slope had often inflected before the emergence of ECG evidence for myocardial ischemia, which provides the possibility to identify ischemic events in advance (16). Therefore, keeping strict observation of the ΔVO_2_/ΔWR slope shape in exercise testing is of great value to ensuring exercise safety and identifying cardiovascular events in exercise assessment.

VE/VCO_2_ slope refers to the ratio of ventilation per minute to carbon dioxide output, with a normal value of 20–30. When the ratio of exercise physiological dead space to tidal volume increases, the ventilation-perfusion relationship shows inconsistence, and the slope of the curve increases abnormally. Abnormal elevation of VE/VCO_2_ slope is an important indicator of poor prognosis in patients with cardiovascular disease (17). This study found that VE/VCO_2_ slope > 30 had an important predictive value for cardiovascular risk during exercise assessment in post-PCI patients. At present, VE/VCO_2_ slope is calculated by linear regression of the data before the ventilatory compensation point in exercise testing. The real-time calculation and result presentation of the VE/VCO_2_ slope during exercise will be conducive to the early identification of exercise risk. The results of this study will provide a basis for the software update of relevant testing equipment.

### Validation of the Prediction Model

In this study, the nomogram prediction model was validated, with 1,006 patients with CHD undergoing CPET after PCI selected as the validation cohort. The area under the ROC curve was 0.807 (95% CI: 0.737–0.877), which was only 0.023 lower than the AUC (0.830) validated within the development cohort, indicating the strong ability of the prediction model for identifying CPET-related cardiovascular events in post-PCI patients and the good discrimination ability of the prediction model.

### Application Prospects of the Prediction Model

An example of a nomogram model application was given as follows. A 70-year-old man after PCI, with diabetes, glycosylated hemoglobin 10%, echocardiogram Sm 8 cm/s, ΔVO_2_/ΔWR inflection during exercise testing, and VE/VCO_2_ slope > 30. The above factor scores were summarized according to the nomogram, 29 points (age) + 36 points (glycosylated hemoglobin) + 75 points (echocardiographic Sm) + 38 points (ΔVO_2_/ΔWR slope inflection) + 20 points (VE/VCO_2_ slope > 30) = 198 points. By taking the total score into the nomogram model, it inferred that the probability was approximately 66%.

Previous studies have shown that exercise assessment in patients with CHD is relatively safe (18), but there is insufficient research on exercise risk prediction in patients with CHD. The nomogram prediction model of cardiovascular events in exercise assessment developed in this study has the following application prospects. Proper use of sensitive CPET indicators to carry out a real-time, dynamic, and comprehensive assessment and prediction of cardiovascular events during exercise in people receiving exercised-based rehabilitation will help identify early warning indicators involved in the model before the occurrence of cardiovascular events, and avoid risks by timely terminating exercise testing.

### Research Limitations

This study is a single-center retrospective study. The results may have certain limitations and need further validation and generalization in more medical institutions. In addition, this study did not cover cardiovascular events during exercise rehabilitation in post-PCI patients. Prospective research is needed to explore methods for predicting and early warning of cardiovascular events during follow-up exercise rehabilitation in patients with CHD based on telemedicine equipment.

## Conclusion

This study developed a model for predicting the risk of exercise-related cardiovascular events. Post-PCI patients with older age, unsatisfactory blood glucose control, impaired left ventricular systolic function, oxygen uptake parameter trajectory inflection, and poor ventilation efficiency have a higher risk of cardiovascular events in exercise assessment. The nomogram prediction model performs well in predicting cardiovascular events in the exercise assessment of post-PCI patients. Results of the study will provide a solid basis for timely identifying and avoiding cardiovascular events in patients with CHD after PCI during exercise testing.

## Data Availability Statement

The original contributions presented in the study are included in the article/Supplementary material, further inquiries can be directed to the corresponding authors.

## Ethics Statement

The studies involving human participants were reviewed and approved by the Ethics Committee of Peking University Third Hospital. The patients/participants provided their written informed consent to participate in this study. Written informed consent was obtained from the individual(s) for the publication of any potentially identifiable images or data included in this article.

## Author Contributions

All authors listed have made a substantial, direct, and intellectual contribution to the work, and approved it for publication.

## Conflict of Interest

The authors declare that the research was conducted in the absence of any commercial or financial relationships that could be construed as a potential conflict of interest.

## Publisher’s Note

All claims expressed in this article are solely those of the authors and do not necessarily represent those of their affiliated organizations, or those of the publisher, the editors and the reviewers. Any product that may be evaluated in this article, or claim that may be made by its manufacturer, is not guaranteed or endorsed by the publisher.
