# Supplementary material for: Development and Validation of a Prediction Model for Cardiovascular Events in Exercise Assessment of Coronary Heart Disease Patients After Percutaneous Coronary Intervention
Source: Front Cardiovasc Med. 2022 Apr 26;9:798446. doi: 10.3389/fcvm.2022.798446 (PMC9086511; doi:10.3389/fcvm.2022.798446)
Supplement: Supplementary file 1 [file Data_Sheet_1.docx]

**Table 1 Clinical information of CHD patients in the development cohort and in the validation cohort**

| **Parameters** | **Development cohort** | **Validation cohort** | ***P* value** |
| --- | --- | --- | --- |
| Age (years) | 60.7±10.1 | 60.6±10.2 | 0.503 |
| Male, N (%) | 1151（79.4） | 801（79.6） | 0.919 |
| BMI (Kg/m^2^) | 25.9±3.7 | 25.6±3.4 | 0.497 |
| History of myocardial infarction, N (%) | 417（28.7） | 325（32.3） | 0.835 |
| Complete revascularization, N (%) | 991（68.3） | 719（71.5） | 0.794 |
| NYHA functional class II, N (%) | 185（12.7） | 212（21.0） | 0.143 |
| Hypertension, N (%) | 895（61.7） | 614（61.0） | 0.736 |
| Diabetes, N (%) | 478（37.9） | 277（27.5） | 0.006 |
| Hyperlipidemia, N (%) | 995（68.9） | 670（66.0） | 0.292 |
| Smoking history, N (%) | 689（47.5） | 439（43.6） | 0.108 |
| Family history of CHD, N (%) | 431（29.7） | 314（31.2） | 0.448 |
| Exercise habit, N (%) | 948（66.4） | 710（70.5） | 0.008 |

Abbreviation：BMI, body mass index;


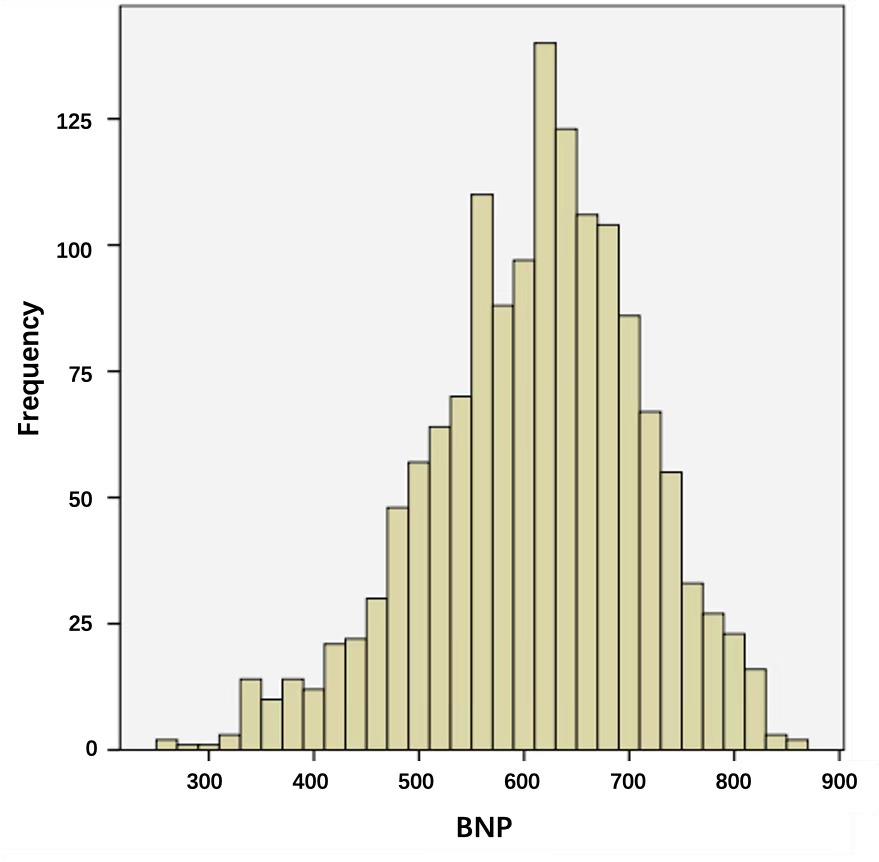


**Figure 2 The histogram of BNP**


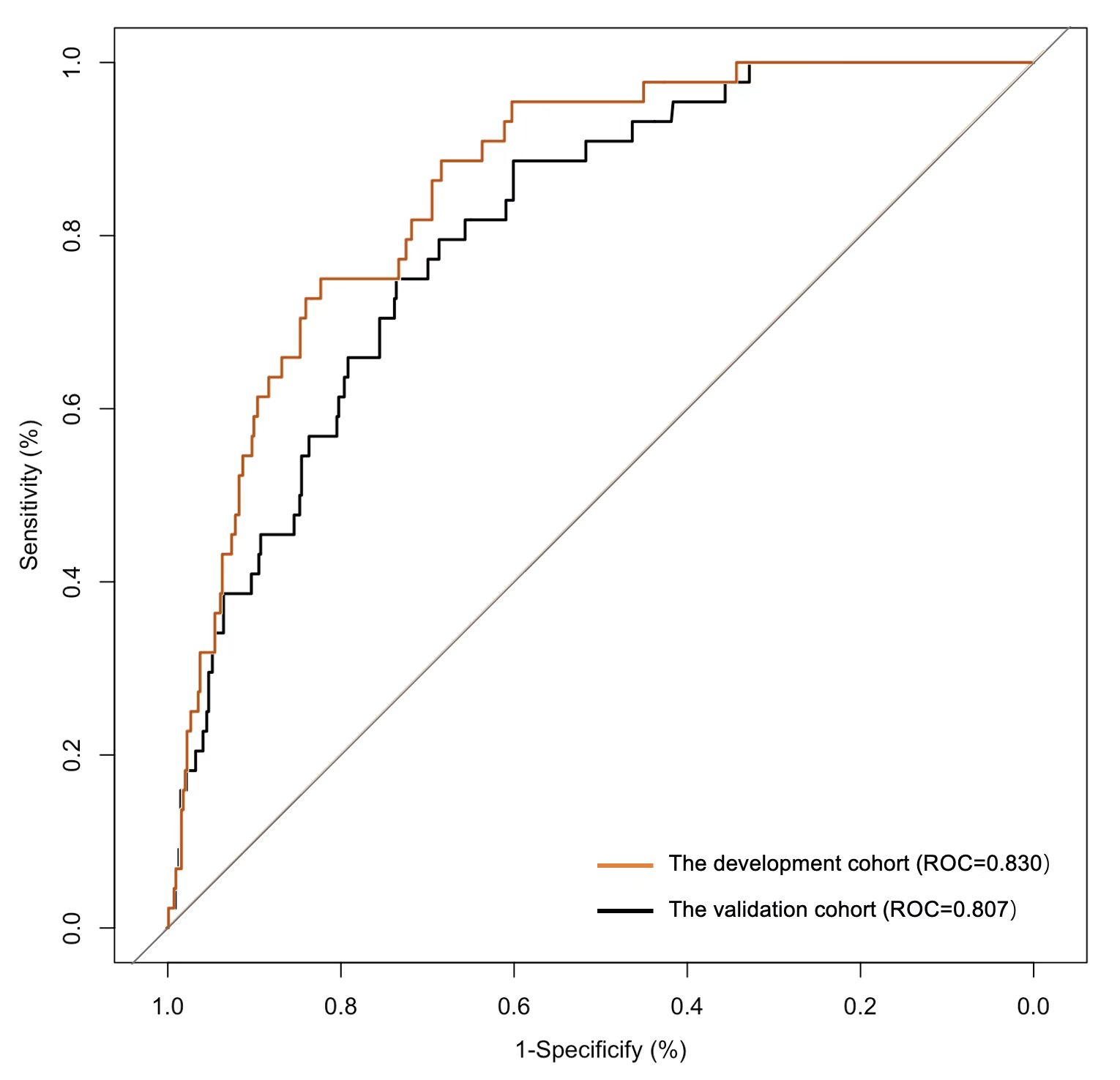


**Figure 2 ROC curve of nomogram model in the in the development cohort and in the validation cohort**
